# Thyroidectomy as Treatment of Choice for Differentiated Thyroid Cancer

**DOI:** 10.1155/2019/2715260

**Published:** 2019-10-13

**Authors:** Dario Giuffrida, Raffaella Giuffrida, Ivana Puliafito, Veronica Vella, Lorenzo Memeo, Caterina Puglisi, Concetto Regalbuto, Gabriella Pellegriti, Stefano Forte, Antonino Belfiore

**Affiliations:** ^1^Department of Experimental Oncology, Mediterranean Institute of Oncology, Via Penninazzo 7, Viagrande, I-95029 Catania, Italy; ^2^IOM Ricerca Srl, Via Penninazzo 11, Viagrande, I-95029 Catania, Italy; ^3^Department of Clinical and Experimental Medicine, Endocrinology Section, University of Catania Medical School, Garibaldi-Nesima Hospital, Via Palermo 636, Catania, Italy

## Abstract

**Background:**

Despite a large amount of data, the optimal surgical management of differentiated thyroid cancer remains controversial. Current guidelines recommend total thyroidectomy if primary thyroid cancer is >4 cm, while for tumors that are between 1 and 4 cm in size, either a bilateral or a unilateral thyroidectomy may be appropriate as surgical treatment. In general, total thyroidectomy would seem to be preferable because subtotal resection can be correlated with a higher risk of local recurrences and cervical lymph node metastases; on the other hand, total thyroidectomy is associated with more complications.

**Methods:**

This is a retrospective study conducted on 359 patients with differentiated thyroid cancer, subjected to total thyroidectomy. Our aim was to correlate clinical and pathological features (extrathyroid tumor growth, bilaterality, nodal and distant metastasis) with patient (gender and age) and tumor (size and histotype) characteristics. Moreover, we recorded postoperative complications, including hypoparathyroidism and laryngeal nerve damage.

**Results:**

In our study, we found a high occurrence of pathological features indicating cancer aggressiveness (bilaterality, nodal metastases, and extrathyroid invasion). On the other hand, total thyroidectomy was associated with relatively low postsurgical complication rates.

**Conclusions:**

Our data support the view that total thyroidectomy remains the first choice for the routine treatment of differentiated thyroid cancer.

## 1. Introduction

Thyroid cancer is the most common endocrine malignancy [1], and it is a heterogeneous disease arising from two different epithelial cell types. Most thyroid cancers are derived from the follicular cells, including papillary thyroid carcinoma (PTC), follicular thyroid carcinoma (FTC), Hürthle cell carcinoma, and anaplastic thyroid carcinoma (ATC). Medullary thyroid carcinoma (MTC) is derived from the parafollicular calcitonin producing cells. PTC and FTC are pooled together under the denomination of differentiated thyroid cancer (DTC). DTC accounts for as high as 90% of all thyroid malignancies [1, 2].

The incidence rate of thyroid carcinoma has been increasing over the past several decades in most countries, but at different magnitudes [3, 4]. According to the American Cancer Society, about 64,300 new cases were diagnosed in the US in 2016 [3]. Explanation for the worldwide increase of thyroid cancer incidence is debated. Some experts believe that the increased number could be due to environmental and lifestyle changes [5, 6]. However, upward trends clearly coincided with the introduction of new and sophisticated diagnostic techniques (ultrasonography, computed tomography, and magnetic resonance imaging), combined with increased medical surveillance and access to healthcare services, that can lead to massive increases in detection of small papillary lesions caused by the large reservoir of asymptomatic, nonlethal disease known to exist in the thyroid gland [2, 7, 8]. Despite the high incidence of this neoplasia, the overall mortality for DTC remains low, at approximately 0.5 cases per 100,000 persons [2], but the risk of local relapse is significant.

The initial surgical treatment for DTC is still controversial. While there is a general consensus regarding initial total thyroidectomy for high-risk patients, conflicting data exist for low-risk patients. It is controversial whether low-risk patients should be treated with total thyroidectomy or lobectomy alone.

In 1996, the American Thyroid Association (ATA) published treatment guidelines for patients with DTC [9], suggesting lobectomy only for low-risk patients with thyroid cancer size less than 1 cm. Other reports proposed that lobectomy for pT1T2 intrathyroid cancer is equivalent in term of survival and recurrence to total thyroidectomy [10, 11]. Over the last 15–20 years, there have been many advances in the diagnosis and therapy of DTC, but clinical controversy still exists. According to the last ATA guidelines [12], total thyroidectomy is recommended when the primary thyroid carcinoma is >4 cm, if there is extrathyroidal invasion, or if regional or distant metastases are clinically present. For tumors that are between 1 and 4 cm in size, either a bilateral or a unilateral thyroidectomy may be suitable as treatment plan. Older age (>45 years), contralateral thyroid nodules, a personal history of radiation therapy to the head and neck, and familial DTC may be criteria for recommending a bilateral procedure.

The aim of this retrospective study was to report our experience in patients with DTC that underwent total thyroidectomy. We reviewed the clinical and pathological features of 359 patients, including extrathyroid tumor growth, bilaterality, and nodal and distant metastases, establishing the frequency of additional tumor foci in the opposite lobe and evaluating any existing association with patient (gender and age) and tumor (size and histotype) characteristics. Moreover, we recorded postoperative complications, including hypoparathyroidism and laryngeal nerve damage.

## 2. Patients and Methods

The records of 359 patients who underwent total thyroidectomy for malignant disease, operated between 1986 and 2012 by two different medical teams sharing comparable surgical skills, were reviewed. Surgical treatment consisted of total thyroidectomy plus pretracheal and paratracheal lymph-node dissection. Unilateral or bilateral lymph-node dissection was performed in 128 (34%) of patients with preoperatively known lymph node metastases.

Total thyroidectomy was carried out in 334 patients as initial treatment (primary total thyroidectomy) on the basis of fine-needle aspiration biopsy (FNAB) diagnosis of malignancy. In the remaining 25 patients, where FNAB was suggestive of malignancy but intraoperative histology could not confirm the diagnosis, a lobectomy with isthmectomy was first performed and the contralateral lobe was excised (completing the total thyroidectomy) after the definitive histological diagnosis became available.

Patients were stratified according to primary tumor size: <1 cm or >1 cm, age: <45 years or >45 years, gender: male or female, histotype: PTC or FTC, and bilaterality.

Postoperative complications including hypoparathyroidism and laryngeal nerve damage were recorded.

Serum proteins and total and ionized calcium levels were measured in all patients to evaluate the presence of temporary or permanent hypoparathyroidism; the patients that required calcium replacement for more than 3 months were classified as having permanent hypoparathyroidism.

Postsurgical laryngoscopy to evaluate recurrent laryngeal nerve function within 3 months after the surgery was performed.

### 2.1. Statistical Analysis

To evaluate differences in the occurrences of specific features, contingency tables were constructed and assessed by the Pearson *χ*^2^ test as test of independence. Patients were stratified for size of tumor (≤1 cm and >1 cm), age at diagnosis (≤45 years and >45 years), gender, and histotype (PTC or FTC), and the occurrences of extrathyroid tumor growth, nodal metastases, distant metastases, and bilaterality were measured.

## 3. Results

### 3.1. Clinical and Histopathological Features vs Patient and Tumor Characteristics

Clinical and pathological features, including extrathyroid tumor growth, bilaterality, nodal and distant metastases, and patient (gender and age) and tumor (size and histotype) characteristics are reported in Table 1.

Sixty-four out of the 359 included patients were men (48%), and 295 (82%) were women; among these, 176 (49.02%) were older than 45 years. Concerning tumor characteristics, the size of the largest tumor was less than 1 cm in 154 patients (42%) and the PTC histotype was present in 306 cases (85.24%), while the remaining 53 patients exhibited follicular histotype (14.76%) (Table 1).

At the time of initial diagnosis, 103 out of 359 (28.7%) patients showed extrathyroid tumor growth. The presence of bilateral differentiated thyroid cancer was found in 23.7% (85 out of 359) of patients. Nodal metastases were found in 128 patients (35.7%), while distant metastasis in 26 (7.2%) patients (Table 1).

### 3.2. Association between Clinical and Histopathological Features and Patient and Tumor Characteristics

To assess potential association between clinical and pathological features (extrathyroid tumor growth, bilaterality, nodal and distant metastases) indicating cancer aggressiveness and patient (gender and age) and tumor (size and histotype) characteristics, contingency tables have been assessed for independency using the Pearson *χ*^2^ test as reported in Table 1 and detailed in Table 2.

In particular, extrathyroid tumor growth was more frequent in tumors >1.5 cm (34.1% vs 21.4%) and in patients with PTC compared to FTC histotype (29.7% vs 22.6%); however, none of these differences was statistically significant (*P* > 0.05, Tables 1 and 2).

The occurrence of bilateral differentiated thyroid cancer was found in 23.7% of all patients (Table 2). Specifically, 43 out of the 154 (27.9%) microcarcinomas (<1 cm) and 42 out of the 205 (20.5%) carcinomas greater than 1.5 cm presented contralateral cancer. In both groups, the frequency of bilaterality was similar and surprisingly high, but no significant association between the occurrence of bilaterality and size of tumor (*P*=0.2452) was found. The same was for age (*P*=0.4432) and gender (*P*=0.2924) (Tables 1 and 2).

On the contrary, the tumor histotype significantly affected almost all the clinical and histopathological endpoints considered, specifically bilaterality and both nodal and distant metastases (Tables 1 and 2).

In particular, 80 out of 306 of PTC patients presented contralateral cancer, whereas for FTC patients, the incidence of bilaterality was significantly lower, 5 out of 53 (PTC 26.1% vs FTC 9.4% 5, *P*=0.048; Table 2). In the same way, lymph node involvement was present in 35.7% of cases, particularly, in 119 out of 306 (38.9%) PTC patients compared to 9 out 53 (17%) of FTC patients (*P*=0.03583; Table 2). Instead, the incidence of distant metastasis was significantly higher in FTC patients (17%, 9 out 53) compared to PTC patients (5.6%, 17 out 306) (*P*=0.01717; Table 2).

A significant association between the presence of nodal metastasis and the age of patients was also found. In fact, 47.5% (87/183) of young patients (<45 years) presented nodal metastasis compared with 23.3% (41/176) of older patients (>45 years) (*P*=0.001292; Table 2).

### 3.3. Postsurgical Complications

In this retrospective study, the incidence of postsurgical complications was also recorded.

In particular, permanent hypoparathyroidism was observed in 11 out of 334 (3.3%) patients who underwent primary total thyroidectomy and in 2 out of 25 (8%) who underwent completion total thyroidectomy (Table 3). Only 3 of these 13 patients needed vitamin D in addition to calcium supplementation. Only 14 out 359 (3.9%) patients showed transient hypoparathyroidism, and they were treated with calcium and vitamin D temporarily (up to six months).

In 3 patients, paralysis of one vocal cord due to the neoplasia was ascertained at preoperative laryngoscopy and persisted after surgery; in these patients, laryngeal nerve damage was not considered a postsurgical complication. Additionally, 1 patient required a temporary tracheostomy because of a transient paralysis of both vocal cords. In this case, total thyroidectomy plus dissection of regional lymph nodes and local muscles had been performed because of the large extension of the tumor. Permanent recurrent laryngeal nerve palsy was observed in 3 patients after primary total thyroidectomy and in 1 patient after completion total thyroidectomy (1.1% of all patients; Table 3). However, just 4 patients presented transient laryngeal nerve damage after primary total thyroidectomy (1.1%; Table 3).

## 4. Discussion

Considerable controversy exists regarding the extent of thyroid surgery at the initial operation for differentiated thyroid carcinoma. This is complicated by the lack of randomized, prospective data to provide guidance for selection of the optimal surgical procedure.

The complication rates of surgery, laryngeal nerve injury, and hypoparathyroidism are the main reason to choose lobectomy in DTC patients with primary tumor size ≤1 cm [13, 14]. However, in the hands of an experienced thyroid surgeon, the complication rate of total thyroidectomy is as low as 2% while the incidence of recurrent laryngeal nerve palsy is increased in cases of reoperation for recurrences in the contralateral lobe [15–17].

The clinical relevance of more complete resection is recognized in high-risk patients and in presence of advanced disease. However, multiple aspects of postsurgical treatment and follow-up are facilitated by more extensive thyroidectomy, even in low-risk patients. The complete thyroid resection allows the use of radioiodine for ablation of residual microscopic disease and subsequent use of thyroglobulin as tumor marker in recurrence monitoring. Nevertheless, several studies have shown no survival benefit from more extensive thyroidectomy in low-risk patients [18, 19]. Noguchi et al., from an analysis of 867 patients affected by papillary thyroid microcarcinoma (PTMC), concluded that total thyroidectomy is not necessary [20]. However, some PTMCs may have an aggressive behaviour and can cause local regional recurrences and cervical lymph node metastases [21, 22]. Other studies demonstrate that unilateral lobectomy was not associated with higher mortality rates, but it was associated with a significantly higher risk of local recurrences or lymph node metastases compared to patients with bilateral surgery [23], supporting the view that bilateral lobal resection probably represents a preferable initial surgical approach to patients with low-risk PTC.

Moreover, the rate of contralateral papillary thyroid carcinoma discovered in completion thyroidectomy or total thyroidectomy specimens is reported to range from 13 to 56%. In a recent study, Pitt et al. [24] reported that the rate of contralateral PTC foci was independent from the primary tumor size and that multifocal disease in the ipsilateral lobe of the primary tumor was a risk factor for contralateral tumor.

Koo et al. [25] found that 16% of 132 patients with clinically unilateral PTMC had occult contralateral carcinoma and concluded that multifocality of the primary carcinoma in the unilateral lobe and the presence of nodules in the contralateral lobe by preoperative evaluation can help to predict the presence of an occult contralateral papillary carcinoma. Pasieka et al. [26] reported that multifocal disease in the primary resected lobe was associated with a high incidence of contralateral tumor. Pacini et al. [27] studied 182 patients with PTC who underwent completion thyroidectomy and found that 44% of the patients had one or more PTC foci at histology. Moreover, they demonstrated that the frequent bilaterality of PTC is not correlated with the prognostic categories of low- or high-risk tumor. Therefore, they suggest performing total thyroidectomy in all patients with PTC diagnosed before surgery and completion thyroidectomy for those initially subjected to limited surgery [27].

In our series, the overall incidence of a bilateral tumor was 23.7%. Interestingly, in patients with microcarcinomas, the occurrence of bilaterality was higher (27.9%) compared to those with PTC >1 cm (20.5%), and although such difference was not statistically significant, it showed a well-defined trend. The only statistically significant difference in bilaterality was found by comparing papillary and follicular histotypes (26.1% vs 9.4%, *P* < 0.05). Indeed, in PTC, two or more separate foci in the thyroid gland frequently occurs, and multifocality is often associated with a high rate of contralateral tumor [26, 28].

The incidence of extrathyroid tumor growth was also rather high in our patients (28.7%), with no statistically significant correlation with all the clinical and histopathological features (sex, age, size, and diagnosis).

Nodal metastases were present in 1 out of 3 patients (35.7%). In particular, their occurrence was significantly much higher in PTC than in FTC histotype (38.90% vs 17.00%; *P*=0.03583), and, in younger patients (<45 years 47.5% vs >45 years 23.3%; *P*=0.001292).

On the contrary, in line with previous studies [29], distant metastases were present more frequently in patients with FTC than in PTC (FTC 17% vs PTC 5.6%; *P* < 0.05).

The main reason why lobectomy seems to be preferable to total thyroidectomy is that total thyroidectomy is more frequently associated with postoperative complications, including hypoparathyroidism and laryngeal nerve damage. Nevertheless, these complications have a low incidence in expert hands [30]. In fact, permanent hypoparathyroidism has been reported to range from 0 to 5.3% [31, 32]. Jeannon described a 9.8% of laryngeal nerve palsy incidence during postoperative time, which decreases up to 2.3% during the follow-up in all patients, because of the recovery of nerve function [33].

Our present study confirms that the rate of these two postsurgical complications is low. In our series of 359 total thyroidectomies managed by two different medical teams with similar surgical skills, permanent hypoparathyroidism was observed in 3.3% of patients after primary thyroidectomy and in 8% of patients that underwent completion thyroidectomy, while 13 (3.9%) patients showed transient hypoparathyroidism. Noteworthy, hypoparathyroidism mostly occurred in patients presenting extensive extrathyroidal tissue involvement by the tumor or in patients who underwent secondary thyroidectomy. Permanent recurrent laryngeal nerve palsy was observed in 4 out of 359 patients (1.1% of all patients) after total thyroidectomy.

## 5. Conclusions

In conclusion, in our experience, considering the high incidence of pathological features indicating cancer aggressiveness (bilaterality, nodal metastases, and extrathyroid invasion) and, on the other hand, the relatively low postsurgical complication rates, we believe that total thyroidectomy with regional lymph-node dissection remains the first choice for the routine treatment of differentiated thyroid cancer. In fact, although some differentiated carcinomas are limited to one thyroid lobe and slowly growing, most of them are multifocal or metastatic and are better cured by total thyroidectomy. Moreover, the preoperative identification of “low-risk” carcinomas is not accurate since important factors are available only after surgery and especially after total thyroid ablation and cannot, therefore, be used to plan the extension of thyroidectomy.

In addition, in patients subjected to extended thyroidectomy, a reduction of recurrence rate is documented by virtually all studies and, finally, the psychological burden of the patient and the high medical cost following a cancer recurrence must also be taken into account.

## Figures and Tables

**Table 1:** Clinical and histopathological features vs patient and tumor characteristics.

		Extrathyroid tumor growth		Bilateral		Nodal metastases		Distant metastases		*P* value			
Patient and tumor features	Tot. *n*°	*N*°	%	*N*°	%	*N*°	%	*N*°	%	Extrathyroid tumor growth	Bilateral	Nodal metastases	Distant metastases
Female	295	85	28.80	65	22.00	102	36.40	19	6.40	*P*=1	*P*=0.2924	*P*=0.6246	*P*=0.3719
Male	64	18	28.10	20	31.30	26	40.60	7	10.90				
<45 years	183	47	25.70	46	25.10	87	47.50	13	7.10	*P*=0.3985	*P*=0.4432	**P=0** **.0013**	*P*=1
>45 years	176	56	31.80	53	30.10	41	23.30	13	7.40				
<1.5 cm	154	33	21.40	43	27.90	48	31.20	8	5.20	*P*=0.06216	*P*=0.2452	*P*=0.3373	*P*=0.3154
>1.5 cm	205	70	34.10	42	20.50	80	39.00	18	8.80				
PTC	306	91	29.70	80	26.10	119	38.90	17	5.60	*P*=0.5221	**P=0** **.0489**	**P=0** **.0358**	**P=0** **.0172**
FTC	53	12	22.60	5	9.40	9	17.00	9	17.00				
Total	359	103	**28.70**	85	**23.70**	128	**35.70**	26	**7.20**				

**Table 2:** Association between clinical and histopathological features and patient and tumor characteristics.

Extrathyroid tumor growth			Nodal metastases		
Tumor size					
≤1 cm	≥1 cm	*P*=0.06216	≤1 cm	≥1 cm	*P*=0.3373
33/154 (21.4%)	70/205 (34.10%)		48/154 (31.2%)	80/205 (39.0%)	
Age of patients at diagnosis					
≤45 y	≥45 y	*P*=0.3985	≤45 y	≥45 y	**P=0** **.001292**
47/183 (25.7%)	56/176 (31.8%)		87/183 (47.5%)	41/176 (23.3%)	
Gender					
Female	Male	*P*=1	Female	Male	*P*=0.6246
85/295 (28.8%)	18/64 (28.10%)		102/295 (36.4%)	26/64 (40.60%)	
Histotype					
PTC	FTC	*P*=0.5221	PTC	FTC	**P=0** **.03583**
91/306 (29.7%)	12/53 (22.60%)		119/306 (38.9%)	9/53 (17%)	

Distant metastases			Bilaterality		
Tumor size					
≤1 cm	≥1 cm	*P*=0.3154	≤1 cm	≥1 cm	*P*=0.2452
8/154 (5.2%)	18/205 (8.8%)		43/154 (27.9%)	42/205 (20.5%)	
Age of patients at diagnosis					
≤45 y	≥45 y	*P*=1	≤45 y	≥45 y	*P*=0.4432
13/183 (7.1%)	13/176 (7.4%)		46/183 (25.1%)	53/176 (30.1%)	
Gender					
Female	Male	*P*=0.3719	Female	Male	*P*=02924
19/295 (6.4%)	7/64 (10.90%)		65/295 (22%)	20/64 (31.3%)	
Histotype					
PTC	FTC	**P=0** **.01717**	PTC	FTC	**P=0** **.04888**
17/306 (5.6%)	9/53 (17%)		80/306 (26.1%)	5/53 (9.4%)	

**Table 3:** Frequency of hypoparathyroidism and laryngeal nerve damage in patients who underwent total thyroidectomy.

	Primary thyroidectomy (*n* = 334)		Completion thyroidectomy (*n* = 25)	
	*n.*	%	*n.*	%
*Permanent hypoparathyroidism*	11	3.3	2	8
*Transient hypoparathyroidism*	13	3.9	1	4
*Permanent laryngeal nerve damage*	3	0.9	1	4
*Transient laryngeal nerve damage*	4	1.2	—	—

## Data Availability

The retrospective data used to support the findings of this study may be released upon application to the Mediterranean Institute of Oncology Review Board, who can be contacted at IRB Chair: iom@grupposamed.com.
